# Mechanical Properties of Low-Performance Concrete (LPC) and Shear Capacity of Old Unreinforced LPC Squat Walls

**DOI:** 10.3390/ma14237310

**Published:** 2021-11-29

**Authors:** Rami Eid, Avraham N. Dancygier, Ghali Jaber

**Affiliations:** 1 Civil Engineering Department, SCE—Shamoon College of Engineering, Beer Sheva 8410802, Israel; 2 Faculty of Civil and Environmental Engineering, Technion—Israel Institute of Technology, Haifa 3200003, Israel; avidan@technion.ac.il (A.N.D.); 24ghali@gmail.com (G.J.)

**Keywords:** low performance concrete, LPC, walls, mechanical properties, shear strength, existing buildings

## Abstract

Low-performance concrete (LPC) is characterized by its low strength and commonly by the presence of large aggregates. This type of concrete was used for construction of load carrying, commonly unreinforced walls in old buildings. The resistance of these buildings with LPC squat walls (of relatively low height-to-length ratio), to in plane horizontal loads, was experimentally investigated in this study. The low compressive strength of these walls, well below that of standard concrete, requires estimation of the relation between the actual LPC compressive strength and its tensile strength, and identification of their failure mode and corresponding shear capacity when subjected to in plane horizontal loads. In this study, compressive and splitting tensile strengths of authentic LPC specimens were measured, and based on them, a relation between the compressive and tensile strengths is proposed. Then, diagonal compression tests were performed on authentic LPC specimens, as well as specimens made of standard concrete. These tests yielded the expected mode of failure of vertical cracking and their analysis shows that their shear capacity needs to be evaluated based on their tensile strength (rather than the flexural shear capacity of unreinforced concrete beams). Thus, the load-bearing (both horizontal and gravitational) capacity to prevent diagonal tension failure of an unreinforced LPC wall can be evaluated by comparing the LPC tensile strength to the major principal stress caused by the load. Assessment of the tensile strength can be based on the relation between the compressive and tensile strengths proposed in this work.

## 1. Introduction

Assessment of existing concrete buildings requires, as a first step, the knowledge of the mechanical properties of the concrete. When dealing with standard concrete, most of its mechanical properties (e.g., tensile strength and modulus of elasticity) are related by empirical expressions to its compressive strength [1,2]. However, these expressions are not valid for non-standard concrete used to construct old existing buildings. In Israel, many buildings (of up to 3–4 stories) that were constructed before the 1970’s include structural walls made of unreinforced low-performance concrete (LPC) also known as “plum concrete” (PC) [3,4,5]. This concrete is characterized by its low strength and commonly by the presence of large aggregates [6,7] (size of 50–300 mm). LPC walls have a large width (>200∼250 mm) and are part of the gravity force resisting system. On the one hand, these buildings were not designed to have a seismic force resisting system and, thus, the walls were not designed to withstand lateral loading such as that resulted from seismic excitation. On the other hand, due to their considerable width, these elements have relatively large stiffness, and therefore they will attract the induced lateral forces from a future earthquake. Consequently, neglecting the effect of LPC walls in the assessment of an existing building resistance to a seismic action can result in an undesired failure of the walls which may lead to a progressive collapse of the entire building.

Very few studies of the mechanical properties of LPC have been performed [3,5,7]. Many buildings in Israel, among them hospitals and schools, have to undergo structural and seismic assessments to provide the owners and the state officers the important information on the procedures of retrofitting and strengthening of such buildings. To do that, the mechanical properties of LPC should be estimated and determined. Moreover, the presence of large aggregates with a low strength matrix around them gives the structure of LPC walls some resemblance to that of masonry walls. One of the methods to determine the shear strength of masonry walls is by conducting a diagonal compression test, performed on rhombus ∼1×1 m${}^{2}$ specimens [8,9]. The failure mode of the specimens in these tests is similar to that of concrete squat shear walls, namely, a failure which is controlled by diagonal tension [10].

This paper presents an experimental research of LPC mechanical properties and shear capacity of old unreinforced squat walls, made of this concrete and subjected to in plane loads. The research goals were to obtain the compressive–tensile strength relation for LPC, realizing that this relation cannot be taken from standard concrete and to evaluate the shear capacity of these walls. In order to achieve the first goal, compressive and splitting tensile tests were performed. The second goal was achieved by performing diagonal compression tests and by analyzing their results, thus providing an insight into the correlation between the concrete tensile strength and the shear capacity of unreinforced LPC squat walls. The shear capacity (controlled by diagonal tension) is also examined analytically and against well known expressions from the literature [8,9]. According to the findings of this study, the tensile strength of this non-standard concrete can be evaluated as a function of its compressive strength, determined from extracted cylindrical specimens. Subsequently, recommendations are proposed for estimating the tensile strength as well as the shear capacity of LPC squat walls.

## 2. Experimental Program

This study dealt with non-standard concrete (LPC) of which old existing buildings were constructed. Thus, the experimental program had two main goals. The first goal was to obtain the compressive–tensile strength relation. The second goal was to evaluate the shear capacity of LPC squat walls that comprise the gravitational load carrying structural system of these buildings. Therefore, the experimental program consisted of three test types: standard tests of compressive and splitting tensile strengths were performed to obtain the first goal and for the second one, non-standard diagonal compression tests were conducted. The latter tests were adopted from a testing procedure of masonry [9].

**Figure 1:**
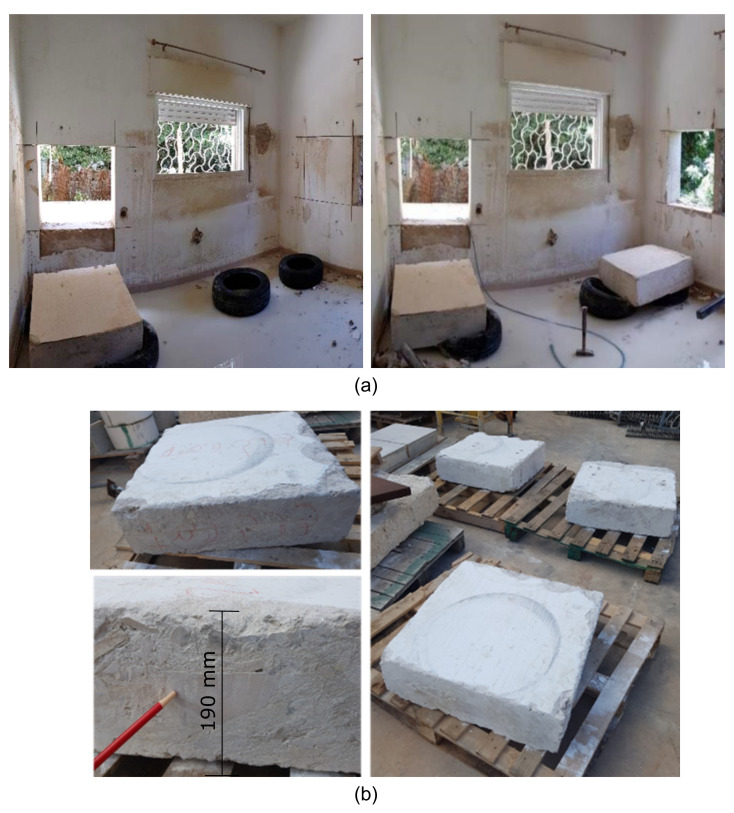
(**a**) Extraction of authentic specimens and (**b**) authentic specimens with large aggregates/stones.

The study of LPC required coping with the challenge of testing specimens that would best represent the properties of this non-standard concrete. Thus, special effort was made to extract authentic specimens from old existing buildings at several different locations as illustrated in Figure 1. LPC specimens were also produced in the laboratory as well as control specimens made of standard low-strength concrete.

The authentic specimens were extracted from five different existing buildings and are represented in the following sections by two letters (representing the places from which they were extracted): LO (extracted from Lotus St., Haifa), BZ (Bnai–Zion hospital), DH (Derech HaYam St., Haifa), TA (Tel Aviv St., Haifa), and SY (Sde Yaakov). The laboratory-reproduced LPC specimens are denoted PCLAB, and those of the control low strength standard concrete are denoted CON80, CON120, and CLAB. Table 1 and Table 2 provide the ingredients of the PCLAB and of the CON80/CON120/CLAB mixtures, respectively.

### 2.1. Compressive Strength

**Figure 2:**
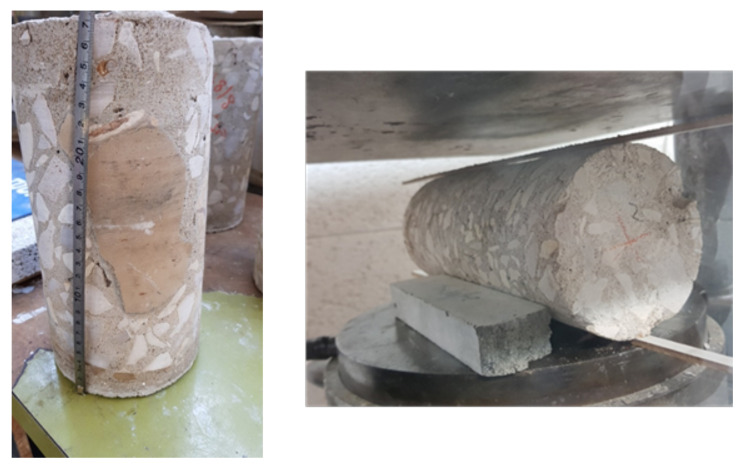
Cylindrical authentic LPC specimens.

The LPC compressive strength, $f_{c}$, was measured by the cylinders that were extracted from the authentic samples. Some of them included the PC characteristic large stones, as can be seen in Figure 2. The results are given in Table 3, which also shows the details of the specimens used to measure them (*H* and *D* are the specimen’s height and diameter, respectively). The resulted maximum measured stresses ($f_{c}$ in Table 3) range from 2.1 to 14.1 MPa. However, due to the effect of H/D ratios smaller than 2, these values were corrected according to ASTM C39/C39M [11] and the corrected values (“corrected $f_{c}$” in Table 3) were used in the following analysis of the results. It is also noted that the scatter in the authentic LPC results is relatively high.

The cylinders (150×300 mm${}^{2}$) used to determine the compressive strength of PCLAB included two types: with and without a large stone/aggregate (70–150 mm). The results are given in Table 4. They show that the inclusion of the large stone/aggregate had a small effect on the average concrete strength (7.6 vs. 7.4 MPa with and without a large stone/aggregate in the specimen). Thus, the average compressive strength of the PCLAB specimens is derived based on the six cylinders of both types and it is equal to $f_{cm}=7.5$ MPa. Table 5 shows the compressive strengths of the control specimens, CON80, CON120, and CLAB, which were made of standard concrete.

### 2.2. Splitting Tensile Tests

Splitting tensile tests were performed for all concrete types (see Figure 2). Note that extraction of the cylindrical specimens from the authentic samples was not simple. This is because these cylinders tended to disintegrate during their drilling, due to the concrete low strength and to the presence of large aggregates/stones, which are typical of this type of concrete. Furthermore, these large stones had to be avoided in the splitting tension specimens in order to minimize their favorable effect on the results.

Table 6, Table 7 and Table 8 show the measured splitting tensile strengths, $f_{\text{sp-exp}}$. Based on the Eurocode 2 (EN 1992-1-1:2004) [1] relation between the axial and splitting tensile strengths, the axial tensile strength $f_{t,sp}=0.9\times f_{\text{sp-exp}}$ was derived from the measured splitting strength, and it is also presented in these Tables. Note that the measured splitting tensile strengths of the authentic specimens showed considerable scatter (coefficients of variation of 16–32%, Table 6) compared with those of the specimens cast in the laboratory.

### 2.3. Diagonal Compression Tests (DCT)

**Figure 3:**
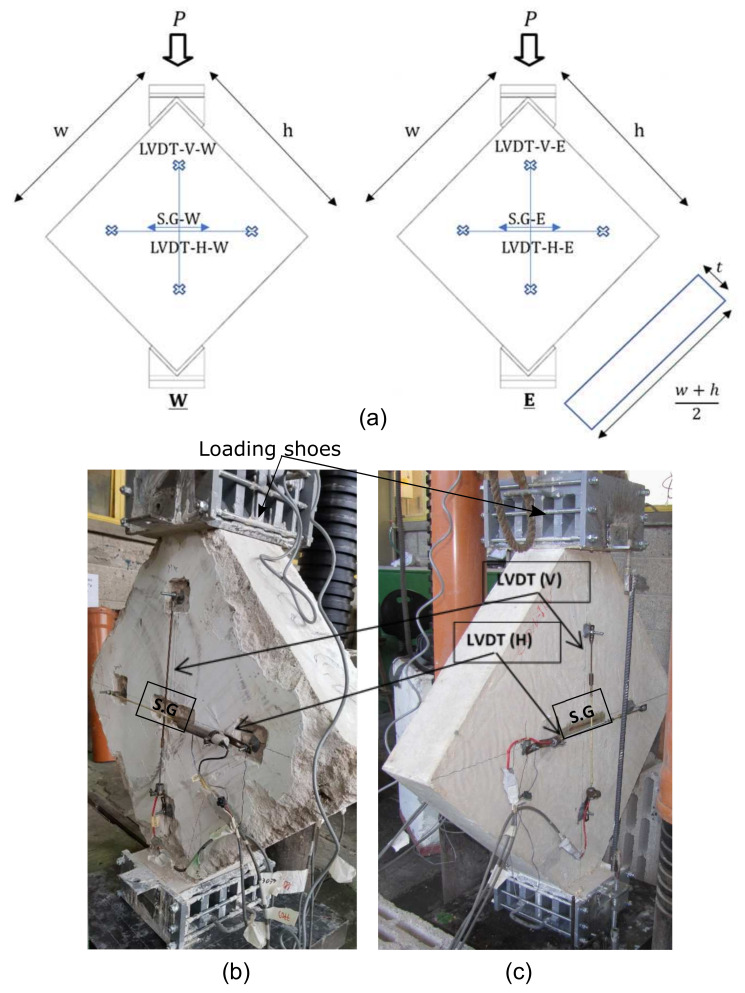
Diagonal compression tests: (**a**) specimens’ details and location of displacement and strain measurement devices, (**b**) authentic, and (**c**) standard concrete specimens.

The presence of large aggregates/stones in the LPC walls binded by low-strength cementitious matrix makes them, from a structural point of view, somewhat similar to masonry walls. Therefore, diagonal compression tests, which are used to examine the shear strength of masonry walls [12], were performed in order to evaluate the shear capacity of LPC walls. Analysis of these test results showed that they are relevant to squat walls, as explained in Section 3. Eight specimens were tested—four authentic LPC (extracted from two sites and denoted LO and DH) and four control specimens cast from low strength, yet standard concrete. The sizes of the latter specimens were 80 × 80 × 15 and 120 × 120 × 15 cm${}^{3}$ to examine any possible size effect, where the two specimen types were denoted CON80 and CON120, respectively. Figure 3 and Table 9 show the details of the specimens.

Load and displacements at the center of the specimens were recorded during the tests, using four (two at each side—horizontal and vertical) Linear Variable Differential Transformers (LVDTs) with a gauge length of ∼500 mm for CON80, LO, and DH specimens and ∼600 mm for CON120 specimens (Figure 3). Moreover, at the same location, two horizontal strain gauges were glued to the specimens, one at each side, with a gauge length of 120 mm for the CON80 and CON120 specimens and 60 mm for the DH specimens (Figure 3). While the strains that were measured from the LVDT records gave an average value (over their gauge length) the strain gauges provided the local horizontal strains at the specimen center.

The load was applied through two steel loading shoes that were attached at the top and bottom sides of the specimens to prevent local failure (Figure 3b). This setup is based on the instructions of ASTM [9] for diagonal compression tests of masonry specimens. A load cell was located between the loading plate of the hydraulic press and the top loading shoe.

## 3. Results

**Figure 4:**
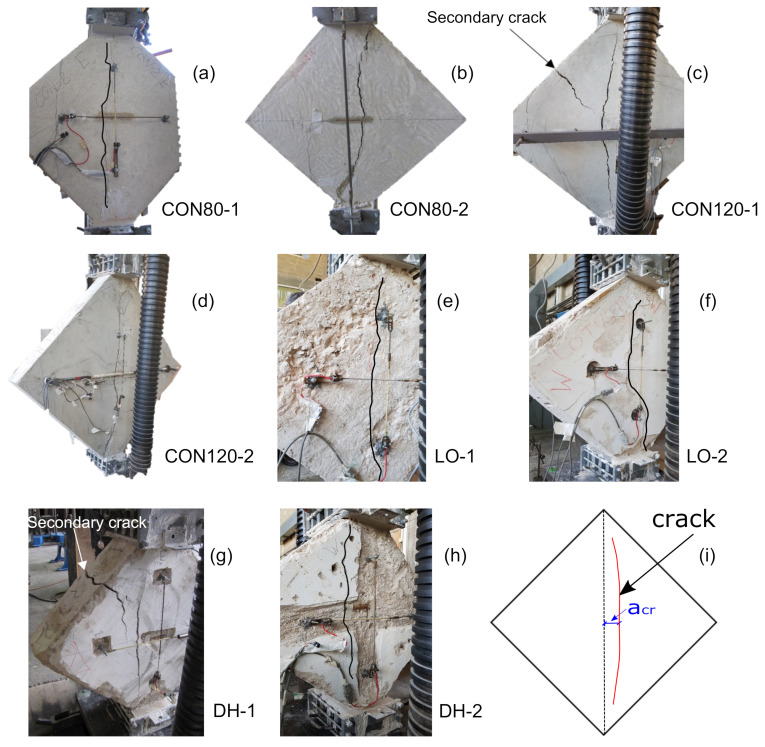
(**a**–**h**) Specimens after diagonal compression tests and (**i**) schematic location of the vertical crack.

All the diagonal compression specimens failed after a vertical crack initiated at their center part and propagated both upward and downward, as expected, see Figure 4. In the authentic specimens the crack developed several centimeters away from the center and a post-test examination of two of them (DH–1 and DH–2) revealed a large stone located at the specimen center.

The maximum load, $P_{max}$, was recorded when the vertical crack was initiated. In two specimens (CON120–1 and DH–1) a secondary crack developed at the top side (with an angle of $45^{\circ }$ from the vertical crack) after the main vertical crack had already propagated (Figure 4c,g). Therefore, the secondary crack, caused by a “knee mechanism” [13] after the specimen’s capacity had been reached, is not relevant to the following analysis of the DCT results. Note that this failure mode characterized by a vertical crack is similar to that excepted in squat unreinforced shear concrete walls [10]. Table 9 gives the maximum load, $P_{max}$, and the measured horizontal distance of the vertical crack from the center of the specimen, $a_{cr}$ (see Figure 4i).

**Figure 5:**
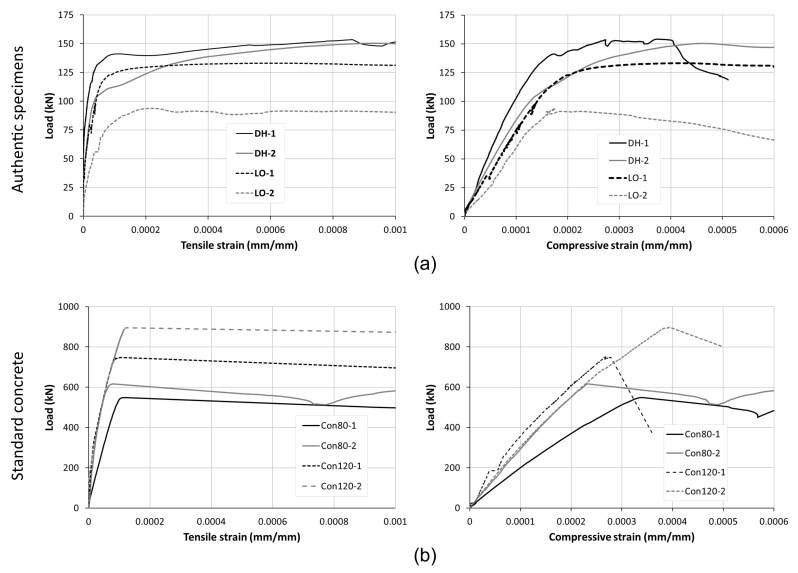
Diagonal compression tests: (**a**) load versus tensile/horizontal strain and (**b**) load versus compression/vertical strain of all specimens.

The response of the specimens was almost linear up to the maximum load (i.e., up to failure). This is evident from the load–strain curves shown in Figure 5. The strains shown in the figure are the tensile/horizontal strain (Figure 5a) and compression/vertical strain (Figure 5b). These strains were calculated from the average readings of the LVDTs attached to both sides of the specimens and they represent averaged strains within the LVDTs’ gauge lengths.

## 4. Analysis of Test Results

Analysis of the current experimental results can be used for provision of the relation between the compressive and tensile strengths of the non-standard, low-performance concrete. Additionally, they provide information regarding the tensile strength of LPC by both splitting tests and diagonal compression tests (DCT). The DCT results also allow to assess the main mechanism that controls the resistance to horizontal loads of existing unreinforced, non-slender LPC walls, as explained in the following sections.

### 4.1. Compressive–Tensile Strength Relations

**Figure 6:**
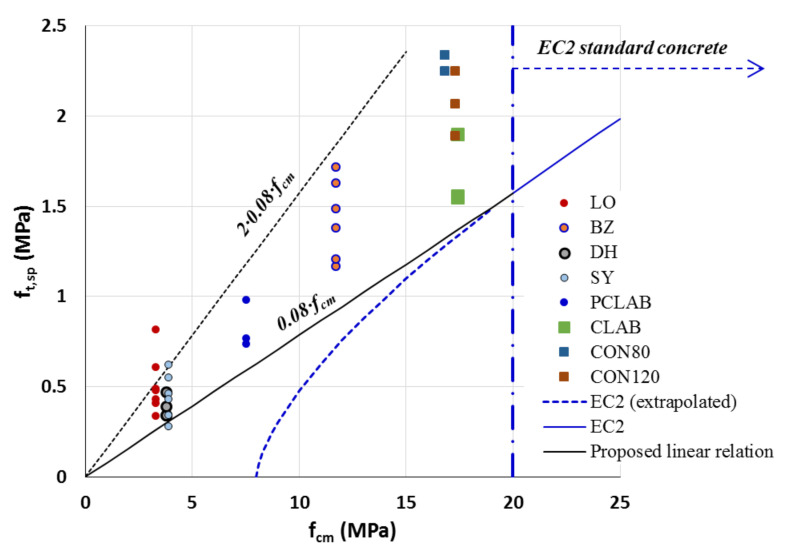
Relationship between axial tensile strength and the mean compressive strength, Reprinted with permission from [14], Copyright 2021 Elsevier.

The measured LPC compressive and tensile strengths have been used to assess the relations between them. Thus, the tensile strengths, $f_{t,sp}$, reported in Table 6, Table 7 and Table 8 were plotted against their corresponding mean compressive strengths, $f_{cm}$, detailed in Table 3, Table 4 and Table 5 and they are shown in Figure 6. The figure includes also the EC2 [1] curve for the mean tensile strength of standard concrete classes, i.e., >C12 (with mean compressive strength of 12+8=20 MPa) given by

(1) $$f_{ct}=0.3\times {\left(f_{cm}-8\left[\mathrm{MPa}\right]\right)}^{2/3}$$

Equation (1) is plotted in Figure 6 with a curved dashed line, which indicates that extrapolation of the code’s relation below the standard strength leads to much lower values than the measured results. Thus, the tensile–compressive strength relation of the authentic LPC concrete is not applicable even for use with the code’s extrapolated relation. Instead, a linear relationship may be more suitable for the LPC’s lower range of compressive strengths, such as the one proposed in the figure and given by:

(2) $$f_{t}\left(\mathrm{LPC}\right)=0.08\cdot f_{cm}\left(\mathrm{LPC}\right)$$

This relationship provides a reasonable (as well as conservative) evaluation of the LPC tensile strength based on the mean compressive strength measured from the samples of existing structures. Note also that Equation (2) merges with the code’s relationship at the lowest standard strength, $f_{cm}=20$ MPa. Examination of the measured strengths shows that their mean value deviates from the proposed linear relationship (Equation (2)) by an absolute error of 33% (Table 6) with a standard deviation (of the errors) of 16%. However, it is noted that all (except one) proposed–to–measured ratios of the tensile strengths fall below 1.0, indicating the conservativeness of Equation (2). This is also illustrated by a straight dashed line in Figure 6 with a slope of 0.16 (depicted in the Figure “$2\times 0.08\times f_{cm}$”) that marks together with the linear relation proposed in Equation (2) a range within which fall most of the values of the measured authentic tensile strengths.

### 4.2. Strength Based on Diagonal Compression Tests

As mentioned above, diagonal compression tests (DCT) were originally developed in order to evaluate the shear capacity of masonry walls. The failure mode of the specimens tested in DCT resembles splitting failure, where a vertical crack initiates at the specimen center and then propagates towards its upper and lower loaded edges (see also Section 3). On the one hand, LPC walls bear a moderate structural resemblance to masonry walls due to the existence of large stones. On the other hand, there is a difference between them, manifested, for example, by the ability to extract cylinders from LPC, on which compressive and tensile strengths can be measured (as discussed above). This is in contrary to masonry, for which DCT tests need to be carried out in order to evaluate their resistance to in-plane loads. Therefore, it was important to examine the correlation between the tensile strength measured by the splitting tests and those that were obtained from the DCT results. In the following, the results of the DCT are analyzed with regards to the concrete tensile strength obtained from the splitting tests. A further consideration is presented of the way these results may correspond to the shear capacity of LPC squat walls.

#### 4.2.1. Tensile Strength

**Figure 7:**
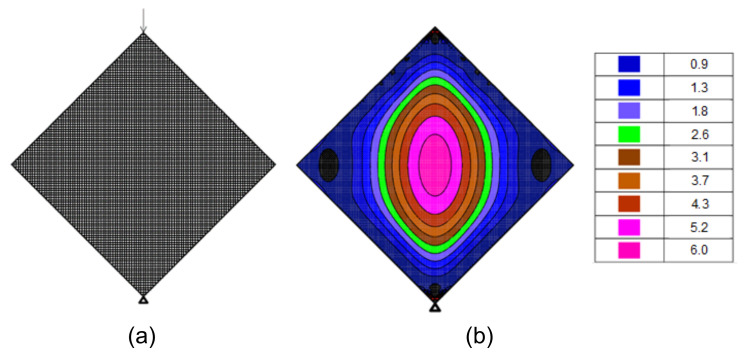
(**a**) Finite element model of a 800×800 mm${}^{2}$ specimen and (**b**) resulted contour of maximum principal stresses [×10 kPa] under a 10 kN load.

The initiation of the vertical crack in the DCT specimens points to maximal principal horizontal tensile stress (perpendicular to the crack), which has been evaluated by

(3) $$\sigma_{t}=\alpha \frac{P}{h\times t}$$

where *P* is the vertical load, and *h* and *t* are the specimen’s width and thickness, respectively (refer to Figure 3 with w=h). For masonry specimens, the coefficient α was evaluated by RILEM [8] and by ASTM [9] to be 0.500 and 0.707, respectively. Given the nearly linear response up to maximum load of the tested specimens (see Figure 5) a linear elastic finite element analysis (FEA) was performed, and it yielded a coefficient of 0.485. Figure 7 describes the 10×10 mm${}^{2}$ elements mesh of a 800×800 mm${}^{2}$ specimen (t=100 mm), analyzed with the STRAP software [15] and the resulted contour of maximum principal stresses under a load of 10 kN.

In addition to confirming the observed failure and the principal stress causing it, the FEA results were used to evaluate the coefficient for the principal stress at a distance $a_{cr}$ away from the specimen centerline, along its mid-height (refer to Figure 4i). This was done by substituting for σ in Equation (3) the major principal stress calculated at a distance $a_{cr}$ from the center (at mid-height). This evaluation followed the observation described in Section 3 of various deviations of the crack initiation points from the specimen’s center. The distances $a_{cr}$ and the corresponding updated coefficient in Equation (3) (depicted here $\alpha_{FE}$) are listed in Table 9. Subsequently, the measured tensile strengths $f_{t,sp}$ (i.e., $f_{t,sp}=0.9\times f_{\mathrm{sp-exp}}$, Table 6, Table 7 and Table 8) were compared in Table 9 with the principal tensile stress under the maximum measured load (Equation (3)) substituting for the coefficient α its values proposed by RILEM [8] and ASTM [9] (depicted $\alpha_{RILEM}$ and $\alpha_{ASTM}$) and values of $\alpha_{FE}$ resulted in from the FEA.

Table 9 shows that for the control, standard concrete specimens (CON80 and CON120, Table 9) application of the RILEM and FEA coefficients yielded calculated-to-measured ratios with mean values of 1.08 and 1.01, respectively, corresponding standard deviations of 0.10 and 0.11 and coefficient of variation (CoV) of 0.09 and 0.11. The ASTM coefficient yielded much higher ratios with a mean value of 1.52. Similarly, for the authentic specimens, mean calculated-to-measured ratios by RILEM and FEA were 1.03 and 0.90 with the same 0.44 CoV, while ASTM yielded a ratio of 1.46.

**Figure 8:**
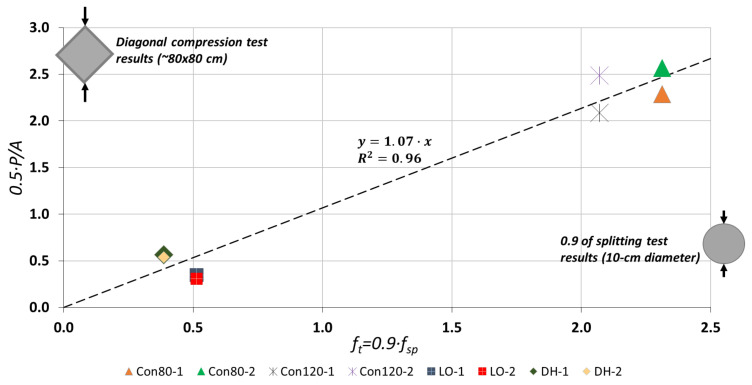
Relationship between tensile strength by diagonal compression test (*Y*-axis) and tensile strength by splitting tensile test (*X*-axis), Reprinted with permission from [14], Copyright 2021 Elsevier.

Therefore, the coefficient proposed by RILEM [8] and those obtained from the FEM analysis yielded the best agreement between the tensile strengths measured in the splitting tensile tests and in the diagonal compression tests, for both standard concrete and LPC. This is also illustrated in Figure 8, which shows a correlation that deviates by no more than 7% between the two strength evaluations, 0.5*P*/*A* (A=h×t) and $0.9\times f_{\mathrm{sp-exp}}$. Thus, these results clearly show that there is good correlation between the tensile strength measured by the splitting tests and those that were obtained from the DCT results. This correlation allows evaluation of tensile strength of existing LPC structural members to rely on splitting tests of extracted cylinders, even if they include large stones.

#### 4.2.2. Structural Shear Capacity

The following analysis demonstrates the relevance of the diagonal compression tests and the concrete tensile strength to the bearing capacity of squat walls to horizontal forces. First, it is verified that the tested specimens’ shear capacity is not controlled by the flexural shear capacity of beams without shear reinforcement. Then, the horizontal force bearing capacity is analyzed with respect to the behavior and capacity of the diagonal compression specimens.

##### Irrelevance of the Flexural Shear Capacity

**Figure 9:**
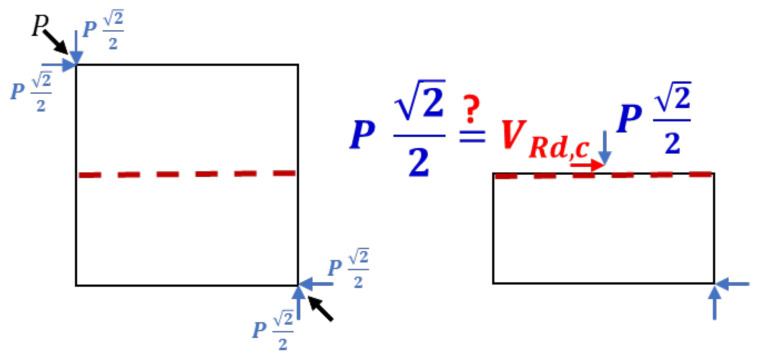
Scheme of the forces that act on a middle section of a diagonal compression specimen, Reprinted with permission from [14], Copyright 2021 Elsevier.

The purpose of this section is to show that the DCT capacity is not dominated by the unreinforced concrete shear resistance, proposed in design codes. To do that, consider the scheme of the forces acting on a middle section of a DCT specimen as illustrated in Figure 9.

Using the EC2 notation for the shear capacity of concrete members without shear reinforcement, $V_{Rd,c}$, it can be seen that under a maximum load *P*, the middle section shown in the figure is acted by a shear force $V_{Rd,c}=P\sqrt{2}/2$ and by an axial compression force, equal to $P\sqrt{2}/2$. Thus, to examine whether the capacity of a DCT specimen is dominated by its shear resistance, $V_{Rd,c}$, calculated from the measured maximum load, can be compared with its value predicted by the code for the standard concrete specimens (CON80 and CON120). As a DCT specimen includes neither shear nor tension reinforcement, its shear capacity is given by the following formula from EC2 (which does not include the longitudinal reinforcement ratio):

(4) $$V_{Rd,c,min}=\left[0.035k^{\frac{2}{3}}f_{ck}^{\frac{1}{2}}+k_{1}\sigma_{cp}\right]b_{w}d$$

where $k=\left(1+\sqrt{\frac{200}{d[\mathrm{mm}]}}\right)\leq 2$, $f_{ck}$ is given in MPa, $k_{1}=0.15$ and $\sigma_{cp}=\frac{P\sqrt{2}/2}{\left(t\cdot h\right)}=\frac{V_{Rd,c,min}}{\left(t\cdot h\right)}$, and noting that for this specimen $b_{w}\cdot d=t\cdot h$. Substituting $\sigma_{cp}$ in Equation (4) and rearranging terms yields:

(5) $$V_{Rd,c}=V_{Rd,c,min}=\left(0.035k^{\frac{2}{3}}f_{ck}^{\frac{1}{2}}\right)t\cdot h\cdot \frac{1}{1-0.15}$$

Substituting these variables and the mean compressive strength of the control specimens 16.8 and 17.3 MPa (Table 8) in Equation (5) yields 37.2 and 37.8 kN, respectively. These values of $V_{Rd,c}$ correspond to $P=37.2\times \sqrt{2}=52.6$ kN and to P=53.4 kN. The measured maximum values of *P* were 548.4 and 616.1 kN for the 80 × 80-cm${}^{2}$ CON80 specimens and 751.3 and 895.8 kN for the CON120 specimens (Table 9). Thus, the predicted $V_{Rd,c}$ capacities are lower by an order of magnitude than the measured capacities. A similar result is obtained by a similar check with the ACI318 equations for shear capacity of concrete without shear reinforcement. Note that the mean compressive strengths were substituted in Equation (5), thus yielding an upper bound of the predicted shear capacities that would have been obtained with smaller characteristic values ($f_{ck}$). Moreover, when squat walls are considered, their shear span-to-depth (a/d) ratios are 1∼1.5, similarly to those of the DCT specimens. Yet, even if the shear capacity $V_{Rd,c}$ (or $V_{c}$ in ACI318), which corresponds to a/d≥3, is increased to account for the short a/d ratios (commonly by applying a factor that relates to a/d, e.g., EC2 [1]), they still do not reach the measured DCT capacities. Instead, the horizontal (shear) force bearing capacity of unreinforced squat shear walls is likely to be determined by the concrete tensile strength.

##### Horizontal Force Bearing Capacity and the Current Results

Considering that old LPC walls do not (or hardly) include any reinforcement, their resistance to horizontal forces, prior to an application of strengthening materials, is controlled by two main types of mechanisms: resistance which is dominated by the wall overall stability (e.g., by rocking) or sliding (commonly near their base) and, in the case of squat walls, by diagonal tensile failure [16]. Once it has been decided to keep an old building with old LPC walls, it is evident that its capacity, related to the former two mechanisms, will be examined. Yet, this examination is out of the scope of the current study, which pertains to the existing LPC squat wall’s resistance to developing diagonal tensile failure. Because this mechanism is controlled by the material tensile strength, the findings reported above can be used to evaluate the resistance of non-slender LPC walls to developing inclined tensile failure under the action of horizontal forces. This evaluation should include calculation of the major principal stress, which is expected to develop under the action of the considered horizontal forces, including the positive effect of any compressive stresses caused by gravity.

Then, the concrete uniaxial tensile strength has to be assessed. This can be done based on the material mean compression strength $f_{cyl,m}$ (measured from cylinders extracted from the building) and the relation proposed in Equation (2), while considering the scatter that was observed in the current study, which can be expressed by the following equation (see Figure 6):

(6) $$f_{t}=0.08\left[-0,+\epsilon \right]f_{cyl,m};\;\text{i.e.},\;0.08\leq \frac{f_{t}}{f_{cyl,m}}\leq 0.08+\epsilon$$

where, based on this study, ϵ is proposed to be equal to 0.08. Once the tensile strength is evaluated, it can be related to the major principal stress under the external loads (both horizontal and gravitational).

## 5. Conclusions

The resistance of old buildings with unreinforced LPC squat walls (of relatively low height-to-length ratio), to in plane horizontal loads, was investigated in an experimental study. LPC is characterized by its low strength and, commonly, by the presence of large aggregates. While some of these old buildings are demolished to allow new construction, there are many one- or two-story buildings, which are still in use and are not planned to be replaced in the foreseen future. These buildings were built with LPC walls that carry the gravitational loads. The low compressive strength of these walls, well below that of standard concrete, requires estimation of the relation between the actual LPC compressive strength and its tensile strength, and identification of their failure mode and corresponding shear capacity, in case of being subjected to horizontal loads. The actual compressive strength is commonly obtained from cylinders that can be extracted from the existing wall, yet because of the inclusion of large stones in these walls, it was not clear to what extent these results or results from splitting tensile tests can be used for this evaluation. The experimental research reported in this paper comprised testing authentic specimens that were extracted from existing buildings at different locations as well as laboratory-reproduced LPC specimens and control specimens made of standard concrete. The main findings of this study, which refer to concrete compressive cylinder strengths up to 14 MPa, are as follows.

- Compressive and splitting tensile strengths of authentic and reproduced LPC specimens were measured.
- Based on these measurements, a relation between the tensile and compressive strengths is proposed, pointing also to the range of possible scatter, which should be examined by the designer when coming to assess the capacity of an existing wall. Note that this new relation is different than that of standard concrete and therefore the findings of this study allow engineers to properly evaluate the mechanical properties of LPC.
- Then, based on the use of relatively large stones in LPC walls, diagonal compression tests (DCT) were performed on authentic LPC specimens, as well as control specimens made of standard concrete. These tests are usually performed on masonry specimens and here they were adopted to study the shear capacity of LPC specimens.
- These tests yielded the expected mode of failure of vertical cracking, caused by principal tension stresses, perpendicular to the external load line of action and corresponding to diagonal tension failure, when the DCT rhombus specimens are considered as cartesian rectangular ones acted by horizontal and normal forces.
- To the best of the authors knowledge, DCT was used in this research for the first time for LPC specimens. For this specimen type, it was found that major principal (tensile) stress is best evaluated by the RILEM approach or by FEM analysis.
- Analysis of the measured maximum load, as well as the specimen dimensions, corresponding to those of squat walls, show that their shear capacity needs to be evaluated based on their tensile strength (rather than the flexural shear capacity of unreinforced concrete beams).
- Thus, the load-bearing (both horizontal and gravitational) capacity to prevent diagonal tension failure of an unreinforced LPC wall can be evaluated by comparing the LPC tensile strength to the major principal stress caused by the load. Assessment of the tensile strength can be based on the proposed compressive–tensile strength relation.

## Figures and Tables

**Table 1:** PCLAB mixture * (laboratory reproduced LPC) in kg/$m^{3}$, Reprinted with permission from [7], Copyright 2021 Elsevier.

Water	196
Cement (CEM I 52.5N)	140
Coarse Aggregate (max. size 25 mm)	830
Coarse aggregate (max. size 19 mm)	220
Natural sand	817

* Not including the large stones.

**Table 2:** Low strength standard concrete mixture in kg/$m^{3}$ [7].

Water	196
Cement (CEM I 52.5N)	206
Coarse aggregate (max. size 19 mm)	1050
Natural sand	760

**Table 3:** Compressive strength of the authentic specimens.

Building	Number of Specimens	*H*	*D*	$f_{c}$	H/D	Correction Factor	Corrected $f_{c}$
		mm	mm	MPa			MPa
		73	74	3.8	0.99	0.87	3.3
		73	74	3.2	0.99	0.87	2.8
		74	74	3.5	1.00	0.87	3.1
		99	94	3.5	1.05	0.88	3.1
		99	94	2.4	1.05	0.88	2.1
		99	94	3.9	1.05	0.88	3.5
LO	11	213	102	2.5	2.09	1.00	2.5
		206	102	2.1	2.02	1.00	2.1
		180	102	5.1	1.76	0.98	5.0
		197	102	3.1	1.93	0.99	3.1
		211	102	5.7	2.07	1.00	5.7
	Average corrected compressive strength, $f_{cm}$ (MPa)						3.3
	Standard deviation (MPa) (Coefficient of Variation)						1.1 (34%)
		223	145	14.1	1.54	0.96	13.5
		234	145	13.7	1.61	0.97	13.3
		157	145	11.5	1.08	0.89	10.2
BZ	6	195	145	10.2	1.34	0.94	9.6
		245	102	11.7	2.40	1.00	11.7
		270	102	12.0	2.65	1.00	12.0
	Average corrected compressive strength, $f_{cm}$ (MPa)						11.7
	Standard deviation (MPa) (Coefficient of Variation)						1.6 (14%)
		182	102	2.9	1.78	0.98	2.8
	3	180	102	4.0	1.76	0.98	4.0
DH		179	102	4.7	1.75	0.98	4.6
	Average corrected compressive strength, $f_{cm}$ (MPa)						3.8
	Standard deviation (MPa) (Coefficient of Variation)						0.9 (23%)
		155	102	10.3	1.52	0.96	9.9
TA	2	152	102	9.6	1.49	0.96	9.2
	Average corrected compressive strength, $f_{cm}$ (MPa)						9.5
	Standard deviation (MPa) (Coefficient of Variation)						0.5 (5%)
		188	102	4.0	1.84	0.99	4.0
		159	102	4.2	1.56	0.96	4.1
SY	4	164	102	3.9	1.61	0.97	3.7
		167	102	3.9	1.64	0.97	3.8
	Average corrected compressive strength, $f_{cm}$ (MPa)						3.9
	Standard deviation (MPa) (Coefficient of Variation)						0.2 (4%)

**Table 4:** Compressive strength of the laboratory-reproduced LPC specimens.

Specimen	Number of Specimens	*H*	*D*	Compressive Strength
		mm	mm	MPa
		300	150	7.7
	3	300	150	6.5
		300	150	8.2
PCLAB		300	150	8.1
	3 (with a large aggregate)	300	150	7.0
		300	150	7.7
	Average compressive strength, $f_{cm}$ (MPa)			7.5
	Standard deviation (MPa) (Coefficient of Variation)			0.7 (9%)

**Table 5:** Compressive strength of the low-strength standard concrete.

Specimen	Number of Specimens	Compressive Strength (MPa)		
		Cylinders	Cubes	Cubes
		150×300 mm${}^{2}$	100×100×100 mm${}^{3}$	150×150×150 mm${}^{3}$
		18.0	25.5	20.5
CON80	9	16.9	23.4	20.3
		15.5	22.3	18.5
Average compressive strength, $f_{cm}$ (MPa)		16.8	23.7	19.8
Standard deviation (MPa) (Coefficient of Variation)		1.25 (7%)	1.6 (7%)	1.1 (6%)
		–	23.1	20.8
CON120	6	–	19.3	20.6
		–	21.4	21.9
Average compressive strength, $f_{cm}$ (MPa)		17.3 ${}^{\left\{1\right\}}$	21.3	21.1
Standard deviation (MPa) (Coefficient of Variation)			1.9 (9%)	0.7 (3%)
		16.4	–	–
CLAB	3	18.0	–	–
		17.9	–	–
Average compressive strength, $f_{cm}$ (MPa)		17.4		
Standard deviation (MPa) (Coefficient of Variation)		0.92 (5%)		

^{1}^ Based on the EC2 [1] relation: $f_{cylinder}$ = 0.82 × $$f_{cube150}$$ 0.82 × 21.1 = 17.3 MPa.

**Table 6:** Splitting tensile strength of the authentic specimens.

Building	No. of Specimens				Splitting Tensile	Axial Tensile	Proposed Relation		
					Strength	Strength			Absolute
		*H*	*D*	$f_{\mathrm{cm}}$	$f_{\mathrm{sp-exp}}$	$f_{t,\mathrm{sp}}{\;}^{\left\{1\right\}}$	$f_{t,\mathrm{prop}.}$ ${}^{\left\{2\right\}}$	$\frac{f_{t,\mathrm{prop}.}}{f_{t,\mathrm{sp}}}$	Error ${}^{\left\{3\right\}}$
		mm	mm	MPa	MPa	MPa	MPa		
		235	102		0.38	0.34		0.77	0.23
		125	102		0.67	0.61		0.43	0.57
		220	102		0.53	0.48		0.55	0.45
LO	7	214	102	3.3	0.54	0.49	0.26	0.54	0.46
		215	102		0.46	0.41		0.64	0.36
		215	102		0.48	0.43		0.61	0.39
		205	102		0.91	0.82		0.32	0.68
	Average splitting tensile strength, $f_{ctm}$ (MPa)				0.57				
	Standard deviation (MPa) (Coefficient of variation)				0.18 (32%)				
		260	102		1.81	1.63		0.57	0.43
		260	102		1.30	1.17		0.80	0.20
		246	102		1.35	1.21		0.77	0.23
BZ	6	200	102	11.7	1.54	1.38	0.94	0.68	0.32
		200	102		1.91	1.72		0.54	0.46
		200	102		1.66	1.49		0.63	0.37
	Average splitting tensile strength, $f_{ctm}$ (MPa)				1.59				
	Standard deviation (MPa) (Coefficient of variation)				0.25 (16%)				
		189	102		0.38	0.34		0.89	0.11
		194	102		0.37	0.34		0.89	0.11
DH	4	182	102	3.8	0.43	0.39	0.30	0.78	0.22
		157	102		0.53	0.47		0.65	0.35
	Average splitting tensile strength, $f_{ctm}$ (MPa)				0.43				
	Standard deviation (MPa) (Coefficient of variation)				0.07 (16%)				
		200	102		0.68	0.62		0.50	0.50
		156	102		0.51	0.46		0.68	0.32
		175	102		0.31	0.28		1.11	0.11
SY	6	165	102	3.9	0.61	0.55	0.31	0.57	0.43
		177	102		0.37	0.34		0.92	0.08
		162	102		0.48	0.43		0.73	0.27
	Average splitting tensile strength, $f_{ctm}$ (MPa)				0.49				
	Standard deviation (MPa) (Coefficient of variation)				0.14 (29%)				
						Mean (MPa)		0.68	0.33
						Standard deviation (MPa)		0.18	0.16
						Coefficient of variation		0.26	0.47

^{1}^$f_{t,sp}=0.9\times f_{\mathrm{sp-exp}}$ (EN 1992-1-1:2004) [1]. ^{2}^ Equation (2). ^{3}^ Absolute error = $|1-\frac{f_{t,prop.}}{f_{t,sp}}|$.

**Table 7:** Splitting tensile strength of the laboratory reproduced LPC specimens.

Specimen	No. of Specimens				Splitting Tensile	Axial Tensile	Proposed Relation		
					Strength	Strength			Absolute
		*H*	*D*	$f_{\mathrm{cm}}$	$f_{\mathrm{sp-exp}}$	$f_{t,\mathrm{sp}}{\;}^{\left\{1\right\}}$	$f_{t,\mathrm{prop}.}$ ${}^{\left\{2\right\}}$	$\frac{f_{t,\mathrm{prop}.}}{f_{t,\mathrm{sp}}}$	Error ${}^{\left\{3\right\}}$
		mm	mm	MPa	MPa	MPa	MPa		
		300	150		0.82	0.74		0.73	0.27
PCLAB	3	300	150	7.53	0.85	0.77	0.60	0.71	0.29
		300	150		1.09	0.98		0.55	0.45
	Average splitting tensile strength, $f_{ctm}$ (MPa)				0.92				
	Standard deviation (MPa) (Coefficient of variation)				0.15 (16%)				
						Mean (MPa)		0.67	0.33
						Standard deviation (MPa)		0.10	0.10
						Coefficient of variation		0.15	0.29

^{1}^$f_{t,sp}=0.9\times f_{\mathrm{sp-exp}}$ (EN 1992-1-1:2004) [1]. ^{2}^ Equation (2). ^{3}^ Absolute error = $|1-\frac{f_{t,prop.}}{f_{t,sp}}|$.

**Table 8:** Splitting tensile strength of the low-strength standard concrete.

Specimen	No. of Specimens				Splitting Tensile	Axial Tensile
					Strength	Strength
		H	D	$f_{\mathrm{cm}}$	$f_{\mathrm{sp-exp}}$	$f_{t,\mathrm{sp}}{\;}^{\left\{1\right\}}$
		mm	mm	MPa	MPa	MPa
					2.60	2.34
CON80	3	Cubes 70×70×70		16.8	2.60	2.34
					2.50	2.25
	Average splitting tensile strength, $f_{ctm}$ (MPa)				2.57	
	Standard deviation (MPa) (Coefficient of variation)				0.06 (2%)	
		200	100		2.10	1.89
CON120	3	200	100	17.3	2.50	2.25
		200	100		2.30	2.07
	Average splitting tensile strength, $f_{ctm}$ (MPa)				2.30	
	Standard deviation (MPa) (Coefficient of variation)				0.20 (9%)	
		300	150		1.72	1.55
CLAB	3	300	150	17.41	1.73	1.56
		300	150		2.11	1.90
	Average splitting tensile strength, $f_{ctm}$ (MPa)				1.85	
	Standard deviation (MPa) (Coefficient of variation)				0.22 (12%)	

^{1}^$f_{t,sp}=0.9\times f_{\mathrm{sp-exp}}$ (EN 1992-1-1:2004) [1].

**Table 9:** Diagonal compression tests—specimens’ details and results.

	Details			Measured			Calculated							
					Average ${}^{\left\{1\right\}}$		with $\alpha_{\mathrm{RILEM}}=0.5$		with $\alpha_{\mathrm{ASTM}}=0.707$		with $\alpha_{\mathrm{FE}}$			
Specimen	*w*	*h*	*D*	$P_{\max}$	$f_{\mathrm{sp-exp}}$	$f_{t,\mathrm{sp}}{\;}^{\left\{2\right\}}$	$f_{t,\mathrm{RILEM}}$	$\frac{f_{t,\mathrm{RILEM}}}{f_{t,\mathrm{sp}}}$	$f_{t,\mathrm{ASTM}}$	$\frac{f_{t,\mathrm{ASTM}}}{f_{t,\mathrm{sp}}}$	$a_{\mathrm{cr}}{\;}^{\left\{3\right\}}$	$\alpha_{\mathrm{FE}}$	$f_{t,\mathrm{FE}}$	$\frac{f_{t,\mathrm{FE}}}{f_{t,\mathrm{sp}}}$
	mm	mm	mm	kN	MPa	MPa	MPa		MPa		mm		MPa	
CON80–1	800	800	150	548.4			2.29	0.99	3.23	1.40	60	0.462	2.11	0.91
CON80–2	800	800	150	616.1	2.57	2.31	2.57	1.11	3.63	1.57	50	0.468	2.40	1.04
CON120–1	1200	1200	150	751.3			2.09	1.01	2.95	1.43	60	0.462	1.93	0.93
CON120–2	1200	1200	150	895.8	2.30	2.07	2.49	1.20	3.52	1.70	30	0.478	2.38	1.15
					Mean			1.08		1.52				1.01
					Standard deviation			0.10		0.14				0.11
					Coefficient of variation			0.09		0.09				0.11
LO–1	800	800	240	133.8			0.35	0.68	0.49	0.96	90	0.434	0.30	0.59
LO–2	800	800	190	93.7	0.57	0.51	0.31	0.60	0.44	0.85	80	0.444	0.27	0.53
DH–1	700	700	195	154.1			0.56	1.46	0.80	2.06	110	0.410	0.46	1.20
DH–2	700	700	200	150.4	0.43	0.39	0.54	1.39	0.76	1.96	60	0.462	0.50	1.28
					Mean			1.03		1.46				0.90
					Standard deviation			0.45		0.64				0.39
					Coefficient of variation			0.44		0.44				0.44

^{1}^ See Table 6 and Table 8. ^{2}^ $f_{t,sp}=0.9\times f_{\mathrm{sp-exp}}$ (EN 1992-1-1:2004) [1]. ^{3}^ Measured.

## Data Availability

The data presented in this study are available on request from the corresponding author.
